# Construction and validation of chemoresistance-associated tumor- infiltrating exhausted-like CD8+ T cell signature in breast cancer: cr-TILCD8TSig

**DOI:** 10.3389/fimmu.2023.1120886

**Published:** 2023-03-06

**Authors:** DQ. Cai, Diankui Cai, Yiping Zou, Xumeng Chen, Zhixiang Jian, Mude Shi, Ye Lin, Jueming Chen

**Affiliations:** ^1^ Department of General Surgery, Sun Yat-Sen Memorial Hospital, Guangzhou, China; ^2^ Department of General Surgery, Guangdong Provincial People’s Hospital, Guangdong Academy of Medical Sciences, Guangzhou, Guangdong Province, China; ^3^ Department of Pharmacology, School of Pharmaceutical Sciences, Hunan University of Chinese Medicine, Changsha, China; ^4^ Guangdong ACXEL Micro & Nano Tech Co., Ltd, Foshan, China; ^5^ Medical College of South China University of Technology, Guangzhou, China

**Keywords:** prognosis, chemoresistance, immunotherapy, signature, tumor-infiltrating CD8+ T cell

## Abstract

**Background:**

Accumulating evidence has revealed that CD8+ T cell exhaustion (Tex) results in worse immunotherapy outcomes. However, the molecular functions and mechanisms of action of Tex in chemoresistance needed to be elucidated.

**Methods:**

The populations of tumor-infiltrating CD8+ T cells (TILCD8Ts) in chemoresistant and chemosensitive groups of the GSE25066 dataset were calculated using CIBERSORT. Differentially expressed genes (DEGs) between TILCD8Ts and other immune cells were explored by integrating 16 immune cell datasets downloaded from the gene expression omnibus (GEO) database. Gene ontology (GO)/Kyoto Encyclopedia of Genes and Genomes (KEGG) enrichment, univariate and multivariate Cox regression, and least absolute shrinkage and selection operator (LASSO) regression of TILCD8T-specific upregulated genes were used to construct a chemoresistant TILCD8T signature (cr-TILCD8TSig). Clinical prognostic data, genomic alterations, chemotherapy response, and immunotherapy response were compared between the different cr-TILCD8TSig subgroups in the GSE25066 and the cancer genome atlas breast cancer (TCGA-BRCA) cohorts.

**Results:**

A cr-TILCD8TSig with exhausted features was identified, consisting of seven genes (*TCF7, RARRES3, ARL4C, ITK, CDH3, GZMB*, and *KLRD1*), which were identified from 104 TILCD8Ts-specific DEGs. Our results showed that compared to the cr-TILCD8TSig-low subgroup, the -high subgroup had a poorer distant relapse-free survival (DRFS) in the GSE25066 cohort and worse progression-free survival (PFS) in the TCGA-BRCA cohort. Univariate and multivariate Cox regression analyses also demonstrated that cr-TILCD8TSig was an independent prognostic factor in the two independent cohorts. Furthermore, cr-TILCD8TSig-low patients benefited more from chemotherapy and immunotherapy than cr-TILCD8TSig-high patients. Besides, we found cell transmembrane signal transduction and the ECM may provide the molecular basis for resistance to antitumor agents in the cr-TILCD8Sig-high subgroup. For genomic alterations, we revealed that mutations in PIK3CA, DMD, and APOB were more common in the cr-TILCD8Sig-high subgroup than in the cr-TILCD8Sig-low subgroup. A nomogram was finally constructed with good discrimination and calibration.

**Conclusions:**

cr-TILCD8TSig is a useful tool to independently predict prognosis, chemotherapy response, and immunotherapy outcomes in patients with breast cancer.

## Introduction

Breast cancer is one of the most common malignant cancers and the leading cause of cancer-related deaths in women worldwide (1). Currently, the treatment options for breast cancer, including mastectomy, chemotherapy, radiotherapy, hormonal therapy, and immunotherapy, are based on molecular profiles, such as estrogen receptor + (ER+), progesterone receptor + (PR+), human epidermal growth factor receptor 2 + (HER 2+), and triple negative, clinicopathologic features, tumor stage, and tumor grade. As a classic treatment, chemotherapy still plays an important role in breast cancer therapy. However, chemoresistance remains a major obstacle to effective breast cancer therapy (2, 3). Therefore, more accurate biomarkers are urgently required for efficient prediction, risk stratification, and treatment decisions.

Recently, the diversity of the molecular portraits of cancers, the complexity of constituents including cell components and non-cell ingredients in the tumor microenvironment (TME), and the intricate cross-reactivity between tumor cells and the TME components have become popular in breast cancer studies (4). It has been established that the complexity of the TME constituents contributes to diverse therapeutic responses and various clinical outcomes in breast cancer (5). Of these, tumor-infiltrating lymphocytes (TILs) are widely considered to play critical roles in mediating breast cancer development, progression, and therapeutic response (6, 7). In addition, tumor-infiltrating CD8+ T cells (TILCD8Ts), a key component of TILs, have been proven to be independent prognostic predictors of breast cancer (8). Previously, Ali et al. suggested that a higher proportion of TILCD8Ts in surgical tumors was associated with better clinical outcomes for breast cancer using immunohistochemical staining (9). However, there is a consensus that TILCD8Ts, including naïve T cells, memory T cells, effector T cells, and exhausted CD8+ T cells (Tex), have different functions in various transcription conditions and diverse differentiated statuses, leading to different immunological phenotypes (10–12). Indeed, the full picture and true role of TILCD8Ts in impacting chemotherapy responses in breast cancer are largely unexplored and urgently needed.

In the current study, by analyzing public databases, we constructed a computational framework based on integrating CD8+ T cell-related genes, infiltration features of breast cancer chemoresistance-related TILCD8T cells, and clinical profile analysis to identify the specific expression patterns of chemoresistance-correlated TILCD8Ts (named “cr-TILCD8TSig”). In addition, we systematically explored the molecular characteristics, genetic variants, chemotherapy and immunotherapy response features, and the potential clinical application of cr-TILCD8TSig.

## Materials and methods

### Data acquisition

The cancer genome atlas breast cancer (TCGA-BRCA) cohort dataset, downloaded from ucsc xena on August 19, 2020, was of the HTSeq-count type. Microarray datasets GSE25066, GSE42058, GSE49910, GSE51540, GSE59237, GSE6863, GSE8059, GSE13906, GSE23371, GSE25320, GSE27291, GSE27838, GSE28490, GSE28698, GSE28726, GSE37750, and GSE39889 were downloaded from Gene Expression Omnibus (GEO) repository and collected using the following platforms: Affymetrix Human Genome U133A Array and Affymetrix HG-U133_Plus 2.0 platform. Tumor and healthy samples were acquired from TCGA-BRCA and GSE25066 datasets. The detailed clinical features are listed in **Supplementary Tables S1** and **S2**.

### Tumor infiltration analysis

Tumor infiltration analysis was based on GSE25066 gene expression data, and the proportion of tumor immune cells in the samples was analyzed using CIBERSORT (13). The analysis was performed using the CIBERSORT default parameters. Taxane anthracycline drug resistance was extracted from GSE25066 and the ratio of 22 different types of immune infiltration in drug-sensitive and non-sensitive samples was calculated. In TCGA-BRCA dataset, clinical samples with drug effects were extracted, and 892 valid samples were used for the following analysis (**Supplementary Table S3**). We estimated the sensitivity of the samples to chemotherapeutic drugs to predict if the patients who provided the samples had died after receiving chemotherapy within the effective clinical follow-up period. Specifically, the samples from breast cancer survivors were drug-sensitive and those from patients who died of breast cancer were drug-insensitive. CIBERSORT was used in the TCGA-BRCA dataset in the same way to calculate the ratio columns of 22 different immune infiltration types in drug-sensitive and non-sensitive samples.

### Data preprocessing

To integrate the expression matrices of GSE42058, GSE49910, GSE51540, GSE59237, GSE6863, GSE8059, GSE13906, GSE23371, GSE25320, GSE27291, GSE27838, GSE28490, GSE28698, GSE28726, GSE37750, and GSE39889 into one expression matrix, the Combat function of SVA package was applied to remove batch effect (14). Then the Dplyr package was used to merge the data into one expression matrix according to the gene probe.

### Identification of differentially expressed and prognostic genes

In the expression matrix, we used the R package “limma” to obtain genes with differential expression between CD8T cells and other types of immune cells (P<0.01). We used the same package to obtain genes with differential expression between drug-sensitive and non-sensitive samples (P<0.01). Cox proportional hazards regression analysis was performed to examine the correlation between gene expression and overall survival (OS). The Coxph function of the survival package was used for Cox analysis of the samples and corresponding genes. Cox analysis can be univariate and multivariate. In univariate COX analysis, target genes were treated as independent factors affecting prognosis, and the risk score and significance of each individual gene were calculated. Overlapping genes with differential expression and prognostic value (P<0.05) in the primary cohort were used to construct a prognostic model. In the multivariate Cox analysis, target genes were treated as cofactors that were correlated with each other. By analyzing the hazard ratio (HR) score of each gene, the sum of the HR score and product of the expression level of the corresponding gene was used as a risk value to measure the risk degree of the sample.

### Construction of a prognostic cr-TILCD8TSig and its validation

In the training cohort, the R package “glmnet” and the least absolute shrinkage and selection operator (LASSO) regression were applied to identify the differentially expressed genes (DEGs) and prognostic gene risk signature. Using the risk score calculation formula: risk score = ∑ (gene expression × corresponding regression coefficient), these genes were classified into high- and low-risk groups based on the median score. Based on the Kaplan–Meier (K-M) method, the R packages “survival” and “survminer” were used to evaluate OS. The R package “timeROC” was used to acquire a receiver operating characteristic (ROC) curve, and the area under the ROC curve (AUC) of the risk score, stage, and grade were used to evaluate the accuracy for predicting OS. Principal component analysis (PCA) was used to explore group distributions. Univariate and multivariate Cox proportional hazards regression models were used to evaluate the impact of the risk score on OS.

### Enrichment analysis

The R package ‘clusterProfiler’ was used to perform gene ontology (GO) enrichment analysis and pathway function enrichment analysis for the target genes. The R package ‘clusterProfiler’ was used to perform pathway function enrichment analysis for different genes in GSE25066.

### Tumor mutational analysis

Then, we downloaded TCGA-BRCA mutational data from TCGA and used the R package ‘maftools’ to analyze single nucleotide variation (SNV) of the data in both the high- and low-risk subgroups (15). The copy number variation (CNV) of these data was analyzed using the GenePattern GISTIC2 algorithm.

### Analysis of immunological efficacy

Based on the expression datasets TCGA-BRCA and GES25066, we used the tumor immune dysfunction and exclusion (TIDE) (https://github.com/liulab-dfci/TIDEpy) algorithm to perform different analyses of immunotherapy efficacy.

### Statistical analysis

The R software (v. 4.0) was used to perform a t-test or ANOVA. Statistical significance was defined as P<0.05 or P<0.01. The levels of significance varied from one analysis to another.

## Results

### Observation of TILs in chemoresistant and chemosensitive breast cancer

**Figure 1:**
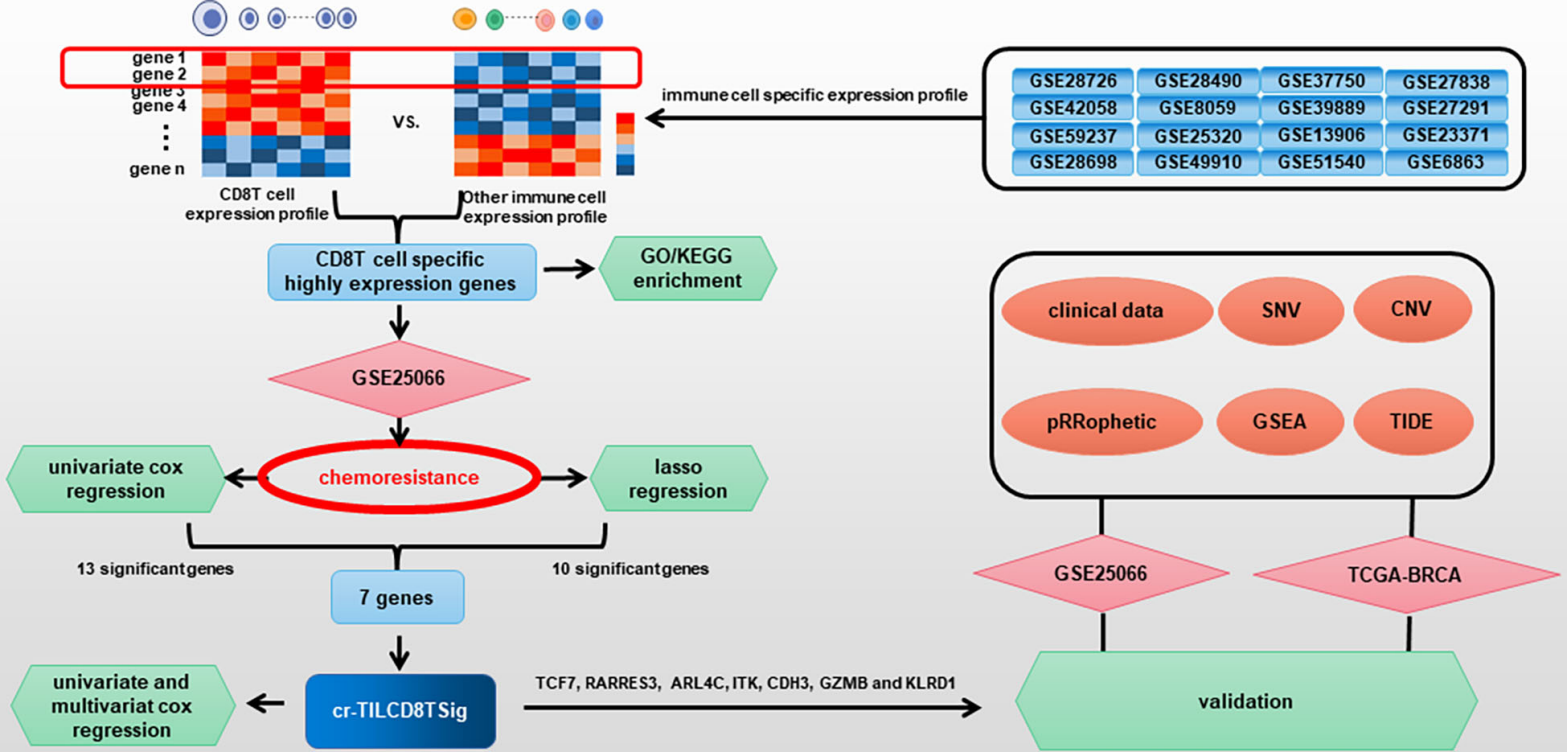
Flow diagram for comprehensive characterization of cr-TILCD8TSig subgroups in breast cancer.

**Figure 2:**
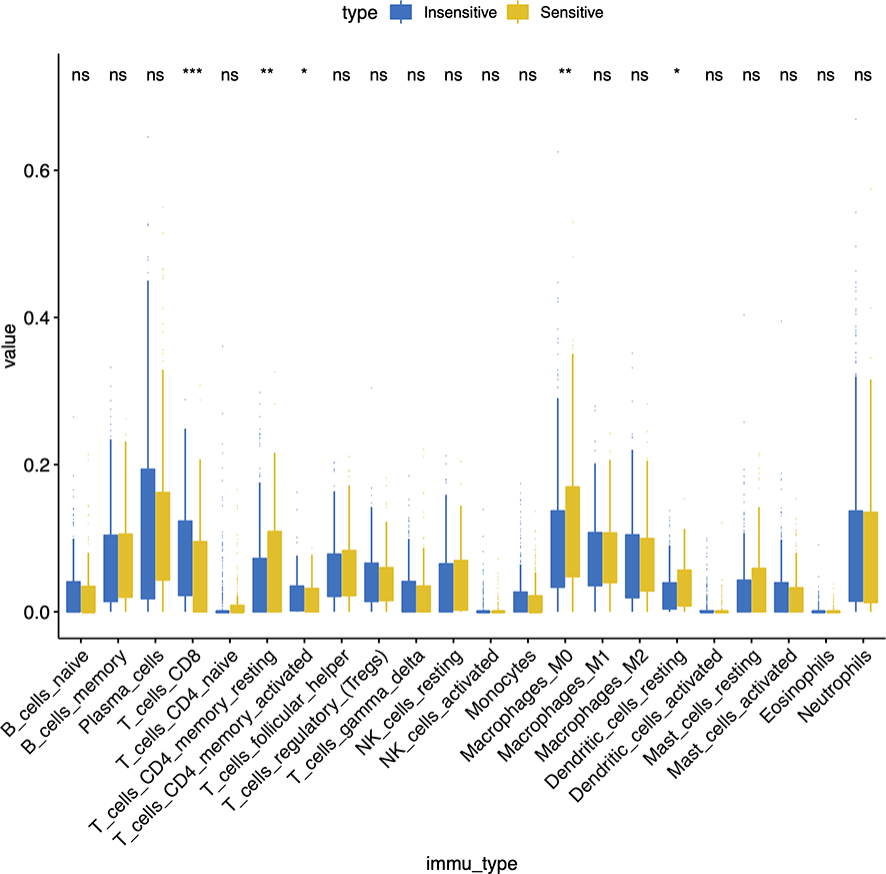
Immune infiltration ratio analysis of taxane anthracycline resistance susceptibility samples in GSE25066.

**Figure 1** described the flowchart of this work. First, we separated the samples of the GSE25066 cohort into chemoresistant and chemosensitive based on the taxane-anthracycline chemotherapy response data. To test whether TILs were involved in the different responses to breast cancer chemotherapy, we estimated the proportions of TILs in the chemoresistant and chemosensitive groups using CIBERSORT. We found that, the proportions of “T_cells_ CD8” and the “T_cells_ CD4_memory_activated” were dramatically decreased, while those of the “T_cells_CD4_memory resting”, “Dendritic_cells_resting”, and “Macrophages_M0” were increased in chemosensitive samples (**Figure 2**, **Table 1**). Especially “T_cells_ CD8” (P=0.0002) seemed to have a more significant effect on chemotherapy responses than “T_cells_ CD4_memory_activated” (P=0.044), “T_cells_CD4_memory resting” (P=0.0089), “Dendritic_cells_resting” (P=0.019), and “Macrophages_M0” (P=0.0079). Therefore, we focused on exploring the relationship between TIL-CD8T and breast cancer chemoresistance.

**Table 1:** Immune cell infiltration types and chemotherapy response in GSE25066 cohort.

immune_type	group1	group2	p	p.adj	p.format	p.signif	method
B_cells_naive	Sensitive	Insensitive	0.577766250095919	0.58	0.58	ns	T-test
B_cells_memory	Sensitive	Insensitive	0.997272307152519	1	1	ns	T-test
Plasma_cells	Sensitive	Insensitive	0.751058881966286	0.75	0.75	ns	T-test
T_cells_CD8	Sensitive	Insensitive	0.000245196108514655	0.00025	0.00025	***	T-test
T_cells_CD4_naive	Sensitive	Insensitive	0.494786591250703	0.49	0.49	ns	T-test
T_cells_CD4_memory_resting	Sensitive	Insensitive	0.0088604458102988	0.0089	0.0089	**	T-test
T_cells_CD4_memory_activated	Sensitive	Insensitive	0.0444887093897064	0.044	0.044	*	T-test
T_cells_follicular_helper	Sensitive	Insensitive	0.298068102001775	0.3	0.3	ns	T-test
T_cells_regulatory_(Tregs)	Sensitive	Insensitive	0.672214992972612	0.67	0.67	ns	T-test
T_cells_gamma_delta	Sensitive	Insensitive	0.56919640335859	0.57	0.57	ns	T-test
NK_cells_resting	Sensitive	Insensitive	0.786865899316298	0.79	0.79	ns	T-test
NK_cells_activated	Sensitive	Insensitive	0.473627380380361	0.47	0.47	ns	T-test
Monocytes	Sensitive	Insensitive	0.265806480949159	0.27	0.27	ns	T-test
Macrophages_M0	Sensitive	Insensitive	0.00789462904578755	0.0079	0.0079	**	T-test
Macrophages_M1	Sensitive	Insensitive	0.882175910362327	0.88	0.88	ns	T-test
Macrophages_M2	Sensitive	Insensitive	0.695164030562406	0.70	0.70	ns	T-test
Dendritic_cells_resting	Sensitive	Insensitive	0.0190341623545046	0.019	0.019	*	T-test
Dendritic_cells_activated	Sensitive	Insensitive	0.356819179100077	0.36	0.36	ns	T-test
Mast_cells_resting	Sensitive	Insensitive	0.0732371979777032	0.073	0.073	ns	T-test
Mast_cells_activated	Sensitive	Insensitive	0.339654172891245	0.34	0.34	ns	T-test
Eosinophils	Sensitive	Insensitive	0.208301786349396	0.21	0.21	ns	T-test
Neutrophils	Sensitive	Insensitive	0.115144807869163	0.12	0.12	ns	T-test

Values were considered statistically significant at p<0.05 (*, p<0.05; **, p<0.01; ***, p<0.001). ns, no significance.

### Identification of TIL-CD8T-specific markers

To screen the CD8-T-cell-specific markers, we analyzed the DEGs between CD8+ T cells and other immune cells by integrating 16 immune cell datasets downloaded from the GEO database. A total of 2219 effective dysregulated genes were evaluated (**Supplementary Table S4**). Of these, 104 were found to be significantly upregulated in CD8 T cells but downregulated in other immune cells (P<0.01, log FC > 1.5) (**Supplementary Figure S1**, **Supplementary Table S5**), and were, thus, considered TIL-CD8T-specific markers.

**Figure 3:**
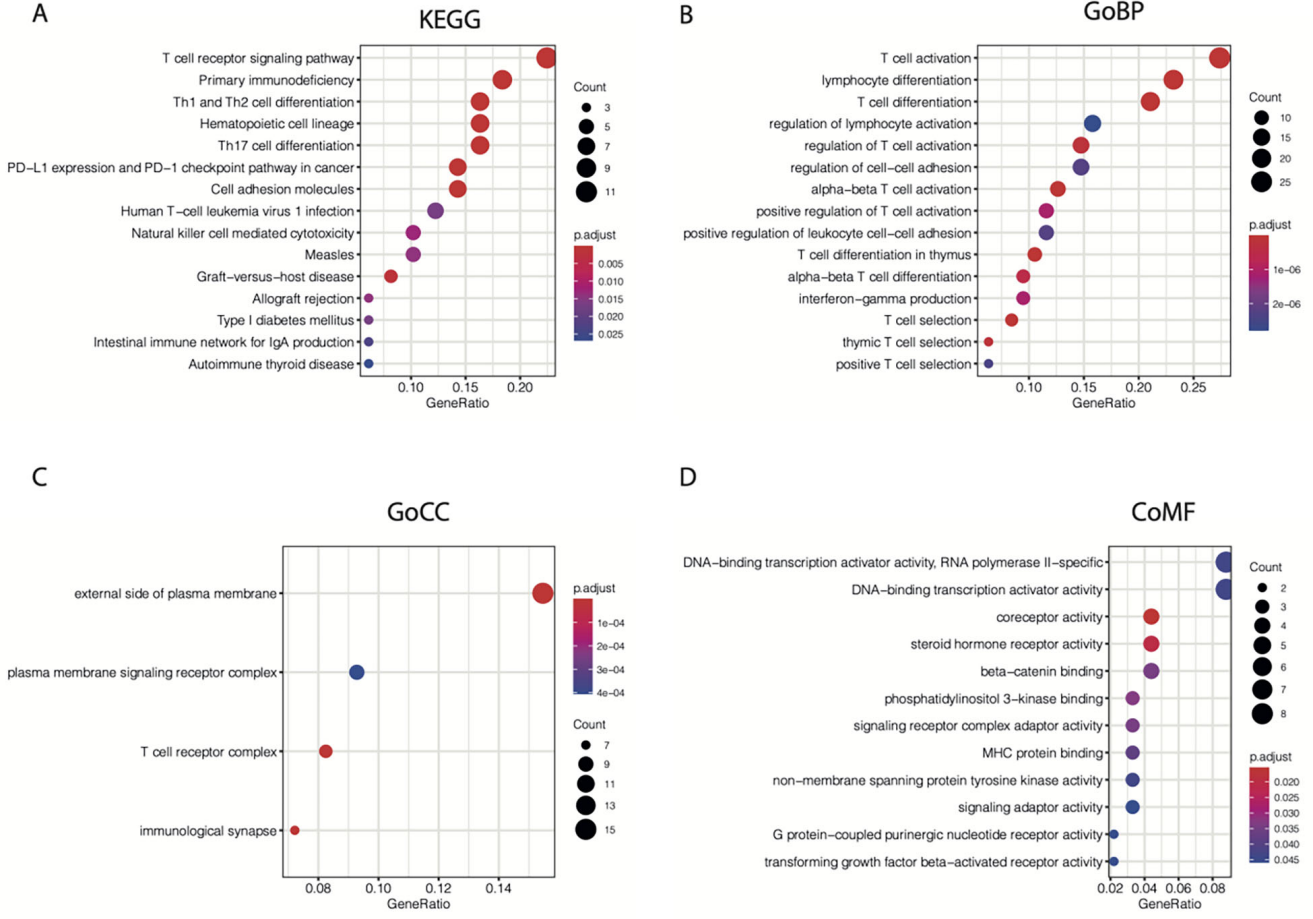
KEGG and GO enrichment results for 104 CD8T-specific markers. 104 CD8T-specific markers were enrolled into KEGG **(A)**, GO-BP **(B)**, GO-CC **(C)**, and GO-MF **(D)** enrichment analysis.

Next, the “cluster Profiler” R package was used for enrichment analysis of the 104 TIL-CD8T-specific markers. We found that the enrichment of the 104 genes in Kyoto Encyclopedia of Genes and Genomes (KEGG) (**Figure 3A**), GO biological processes (**Figure 3B**), GO cellular component (**Figure 3C**), and GO molecular functions (**Figure 3D**) terms were all involved in immunity.

### Construction of a chemoresistant breast cancer associated TIL-CD8T-signatures risk scoring model

**Figure 4:**
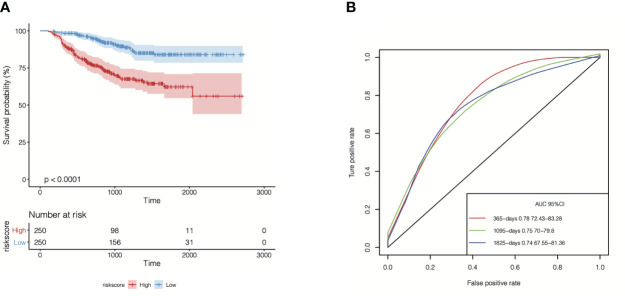
Overall survival analysis of TILCD8TSig in GSE25066. **(A)** Kaplan-Meier analysis of the TILCD8TSig subgroups in GSE25066 cohort. **(B)** ROC analysis of TILCD8TSig on DRFS at 1-,3-, and 5-years follow up in GSE25066 cohort.

Subsequently, we performed a univariate Cox regression analysis to evaluate the correlations between distant relapse-free survival (DRFS) time and the expression levels of the 104 TIL-CD8T-specific markers in breast cancer patients from the GSE25066 cohort (the training set in this study). The results showed that 21 genes were significantly correlated with DRFS time (P<0.05) (**Supplementary Table 6**, **Figure 6A**). Furthermore, the 21 TIL-CD8T specific genes were included in the multivariate Cox regression model, and the risk score, named “TILCD8TSig score,” which was calculated as the sum of the expression values of the 21 TIL-CD8T specific genes multiplied by their relative HR, was constructed and applied to separate the 500 samples of the GSE25066 cohort into TILCD8TSig-high (n=250) and TILCD8TSig-low (n=250) groups based on the median value as a cut-off. K-M analysis showed that, in contrast to the patients in the TILCD8TSig-high group, those in the TILCD8TSig-low group exhibited prolonged DRFS (P<0.0001, **Figure 4A**). The time-dependent ROC analysis also indicated that the AUCs were all greater than 74% at 1-, 3-, and 5-year DRFS in the GSE25066 cohort (**Figure 4B**).

**Figure 5:**
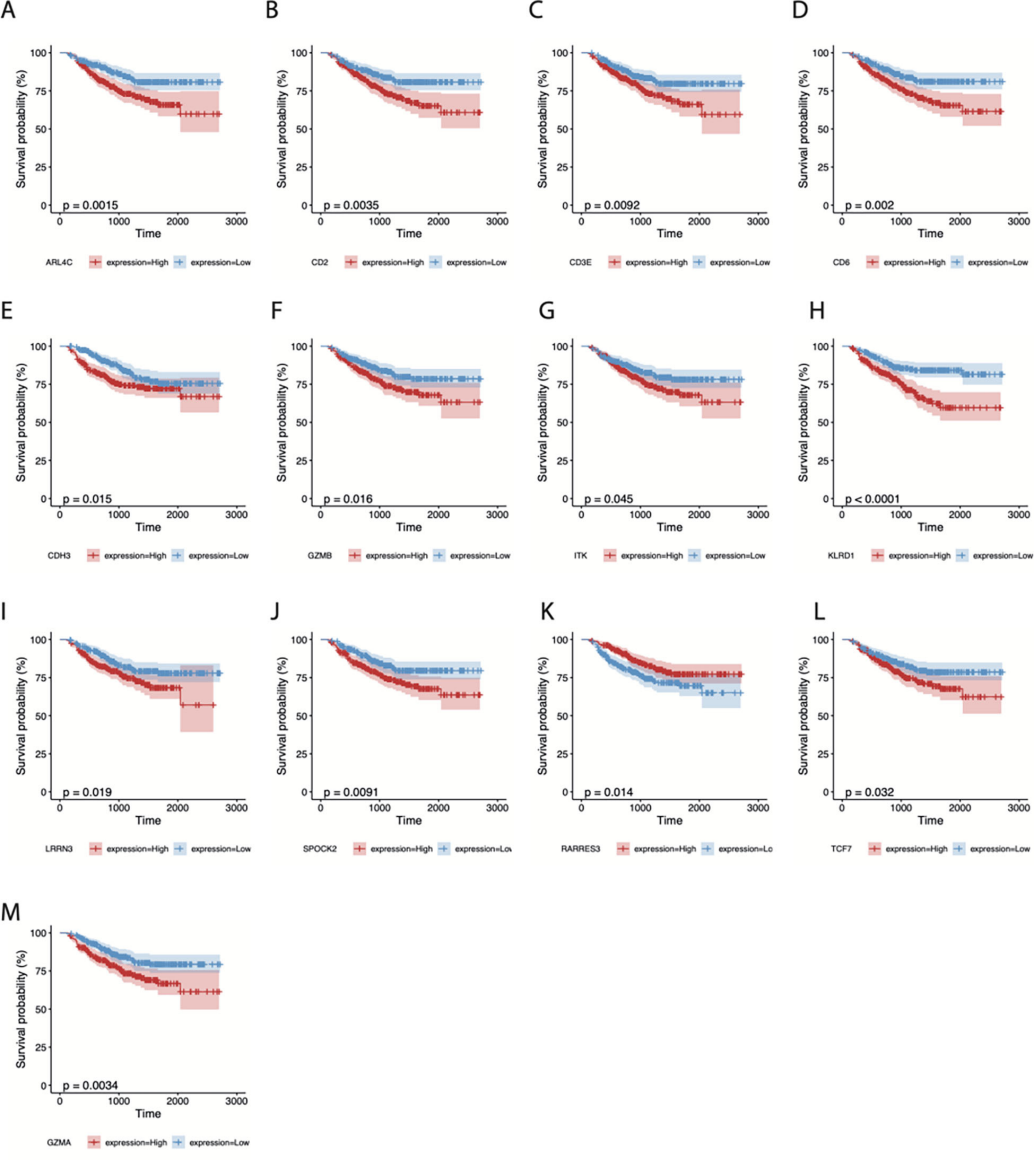
Survival analysis of 13 CD8T-specific high-expression genes. DRFS effects of ARL4C **(A)**, CD2 **(B)**, CD3E **(C)**, CD6 **(D)**, CDH3 **(E)**, GZMB **(F)**, ITK **(G)**, KLRD1 **(H)**, LRRN3 **(I)**, SPOCK2 **(J)**, RARRES3 **(K)**, TCF7 **(L)**, and GZMA **(M)**.

**Figure 6:**
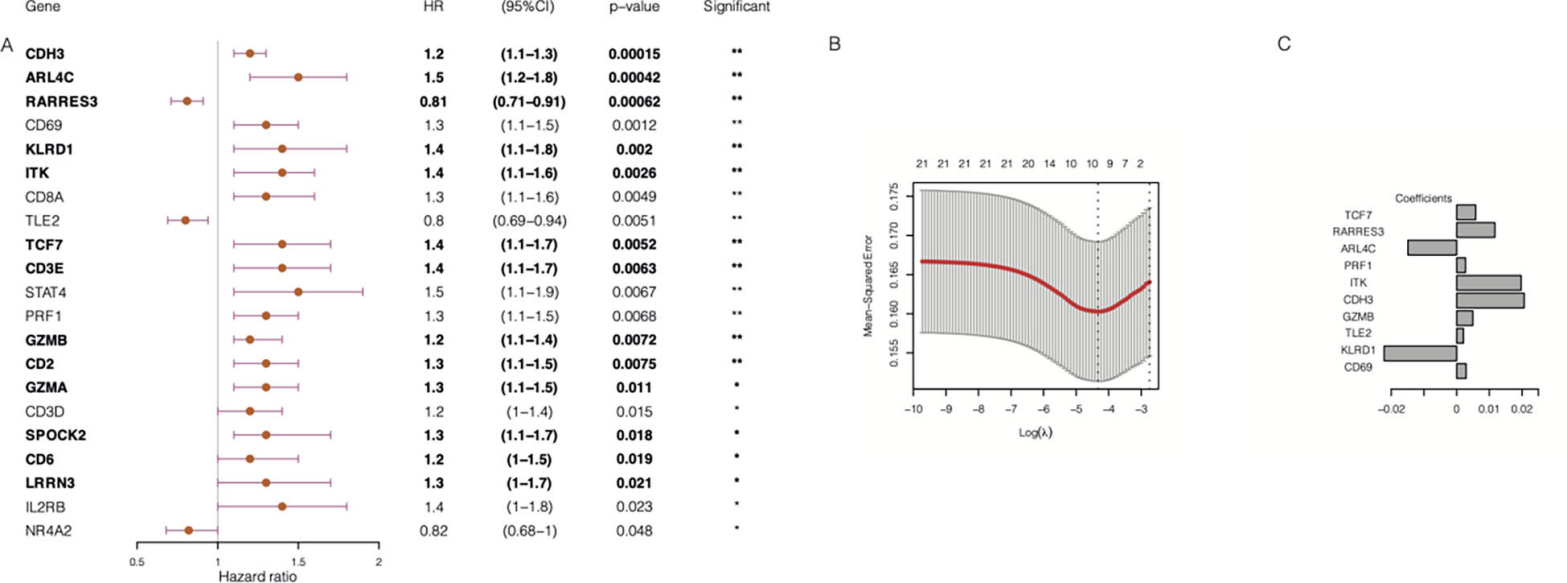
Construction of chemoresistance-associated TILCD8TSig (cr-TILCD8TSig). **(A)** Univariate Cox regression analysis results of 21 genes involved in TILCD8TSig (Bold fonts indicates the 13 genes with significant prognostic value based on Kaplan-Meier analysis.) **(B)** Lasso analysis revealed 10 genes that were significantly prognostically characteristic. **(C)** Coefficient of the 10 genes. Values were considered statistically significant at p<0.05 (*, p<0.05; **, p<0.01).

In addition, 500 samples from the GSE25066 cohort were divided into high- and low-expression groups based on the expression levels of the 21 TIL-DC8T-specific genes using the median expression values as a cut-off. K-M curves revealed that 13 genes (ARL4C, CD2, CD3E, CDH3, CD6, GZMB, ITK, KLRD1, LRRN3, SPOCK2, RARRES3, TCF7, and GZMA) differed significantly in the correlations between their expression levels and DRFS, while 8 genes (CD3D, CD8A, CD69, IL2RB, NR4A2, PRF1, STAT4, and TLE2) had no significant effects (**Figure 5**, **Supplementary Figure S2**, **Supplementary Table S7**). Next, the 21 TIL-DC8T-specific genes were entered into LASSO regression analysis, and 10 (TCF7, RARRES3, ARL4C, PRF1, ITK, CDH3, GZMB, TLE2, KLRD1, and CD69) were found to be significantly related to DRFS (**Figures 6B, C**).

By taking the intersection of the 13 genes revealed using the K-M method and the 10 genes identified using LASSO analysis, seven genes (TCF7, RARRES3, ARL4C, ITK, CDH3, GZMB, and KLRD1) were finally obtained as the chemoresistance-associated TILCD8TSig (cr-TILCD8TSig) for the following analysis. By reviewing the literature in PubMed, we found that TCF7, KLRD1, GZMB, and ITK are related to T cell exhaustion (10, 16). These results imply that cr-TILCD8TSig may exhibit features of T cell exhaustion.

### Evaluation and validation of the cr-TILCD8TSig scoring model for predicting prognosis in the GSE25066 and TCGA-BRCA cohorts

**Figure 7:**
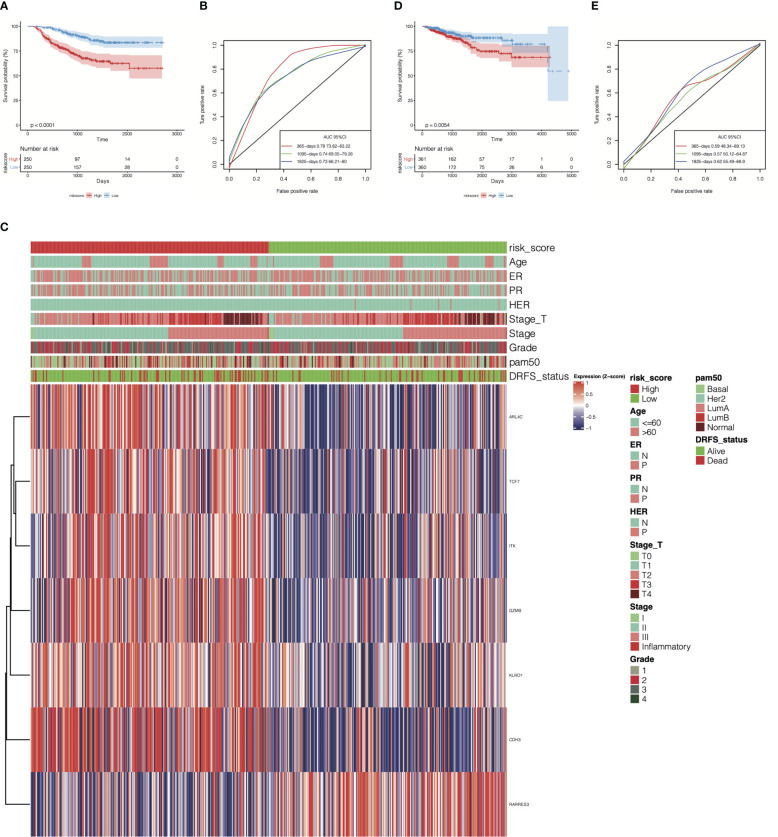
Prognostic energy efficiency analysis of cr-TILCD8TSig scoring models. **(A)** Kaplan-Meier analysis of the cr-TILCD8TSig subgroups in GSE25066 cohort. **(B)** ROC analysis of cr-TILCD8TSig on DRFS at 1-,3-, and 5-years follow up in GSE25066 cohort. **(C)** The cr-TILCD8TSig grouping in the GSE25066 cohort. Age, ER, PR, HER, stage_T, stage, grade, pam50 and survival status are shown as patient annotations. **(D)** Kaplan-Meier analysis of the cr-TILCD8TSig subgroups in TCGA-BRCA cohort. **(E)** ROC analysis of cr-TILCD8TSig on DRFS at 1-,3-, and 5-years follow up in TCGA-BRCA cohort.

To evaluate the efficiency of the cr-TILCD8TSig scoring model used for predicting prognosis in breast cancer patients in the GSE25066 (the training set) and TCGA-BRCA (the validation set) cohorts, a multivariate Cox regression model of the seven cr-TILCD8TSig was used. The results showed that, in the training set, the DRFS was dramatically shortened in the cr-TILCD8TSig-high group compared with that in the cr-TILCD8TSig-low group (P<0.0001, **Figure 7A**). The ROC curves showed that the AUC of cr-TILCD8TSig was over 73% at 1-, 3-, and 5-year DRFS (**Figure 7B**). The clinical features distribution on the cr-TILCD8TSig-high and -low subgroups also indicated that deaths and stage T4 high-grade events occurred more frequently in the cr-TILCD8TSig-high group than in the -low group, whereas age, ER, and HER status did not differ between the two groups (**Figure 7C**). Consistently, the cr-TILCD8TSig score for each patient in TCGA-BRCA cohort was computed and then stratified into two groups, cr-TILCD8TSig-high and -low, according to the median value as a cut-off. Patients in the cr-TILCD8TSig-high group exhibited a poorer progression-free survival (PFS) rate than those in the cr-TILCD8TSig-low group (P=0.0054, **Figure 7D**). The AUC of cr-TILCD8TSig was >50% at 1-, 3-, and 5-year PFS (**Figure 7E**). These results demonstrated that the cr-TILCD8Sig scoring model had a good discrimination ability in the training and validation sets.

**Figure 8:**
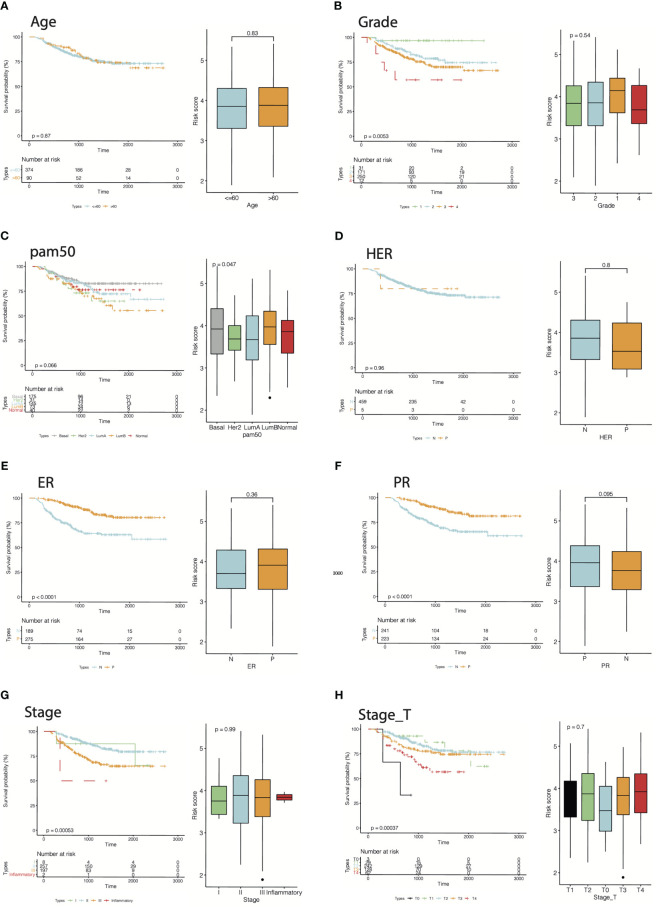
Energy efficiency and stability assessment of cr-TILCD8TSig scoring models. Prognostic significance of cf-TILCD8TSig in GSE25066 cohort stratified by age **(A)**, grade **(B)**, pam50 **(C)**, HER **(D)**, ER **(E)**, PR **(F)**, stage **(G)**, and stage_T **(H)**.

**Figure 9:**
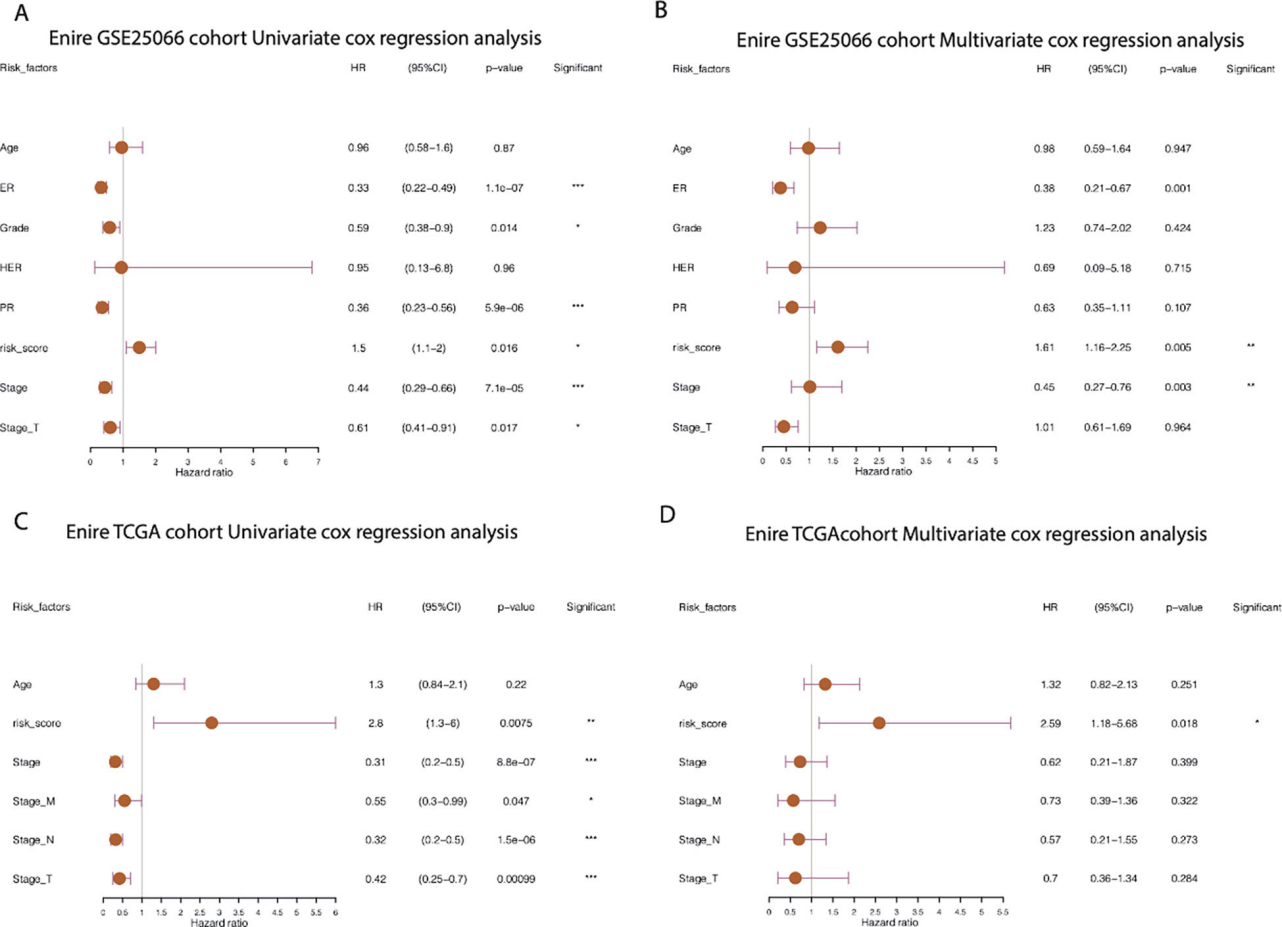
Prognostic independence analysis of cr-TILCD8TSig scoring models. Values were considered statistically significant at p<0.05 (*, p<0.05; **, p<0.01; ***, p<0.001).

Furthermore, by extracting the clinical information of the GSE25066 dataset to examine the robustness of the cr-TILCD8T scoring model, we found that the cr-TILCD8TSig scoring model exhibited significant prognostic differences in groups with different clinical features, such as tumor grade, tumor stage, stage_T, ER status, and PR status (**Figure 8**). Univariate and multivariate Cox regression analyses also proved the independent prognostic value of the cr-TILCD8TSig scoring model in the GSE25066 and TCGA-BRCA cohorts (**Figure 9**).

### Comprehensive analysis of molecular characteristics and genomic alterations between the different cr-TILCD8TSig subgroups

**Figure 10:**
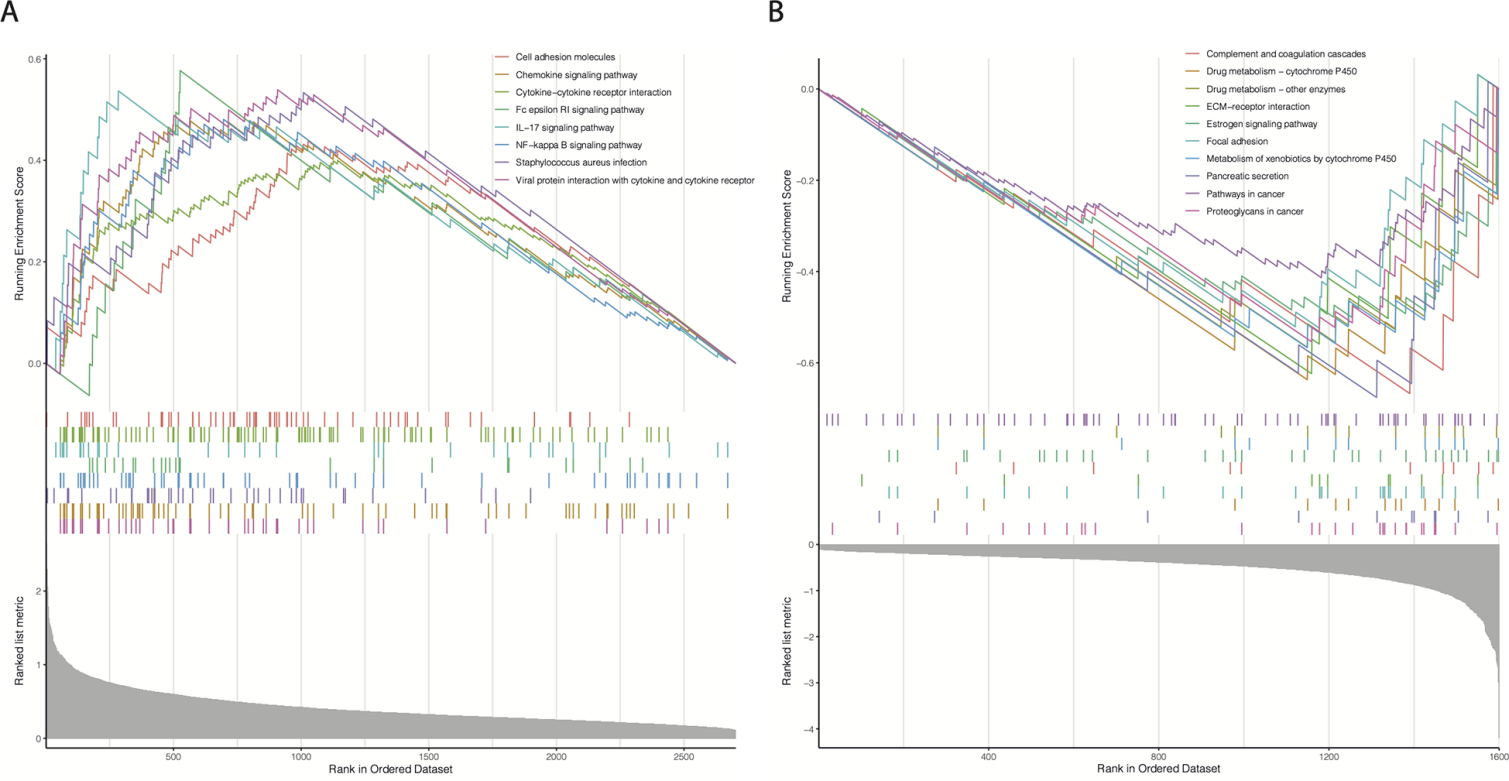
Analysis of GSEA pathway differences between different cr-TILCD8TSig subgroups.

We hypothesized that the prognostic differences between the cr-TILCD8TSig-high and -low subgroups were caused by alterations in the gene transcription levels. To further analyze the molecular characteristics of the different cr-TILCD8Sig subgroups, 4303 DEGs were identified in TCGA-BRCA (P<0.01). A total of 1600 DEGs were significantly upregulated in the cr-TILCD8Sig-low group, whereas 2703 DEGs were upregulated in the cr-TILCD8Sig-high group (**Supplementary Table S8**). GSEA was performed for the KEGG pathway enrichment analysis. The results showed that the gene sets of the cr-TILCD8TSig-high samples were enriched in cell adhesion molecules, chemokine signaling pathways, cytokine-cytokine receptor interactions, and immune response-related pathways (**Figure 10A**), while those of the cr-TILCD8TSig-low samples were mainly enriched in drug metabolism-related pathways and extracellular matrix (ECM)-receptor interaction pathways (**Figure 10B**). These results indicate that cell transmembrane signal transduction and the ECM may provide the molecular basis for resistance to antitumor agents in the cr-TILCD8Sig-high subgroup.

**Figure 11:**
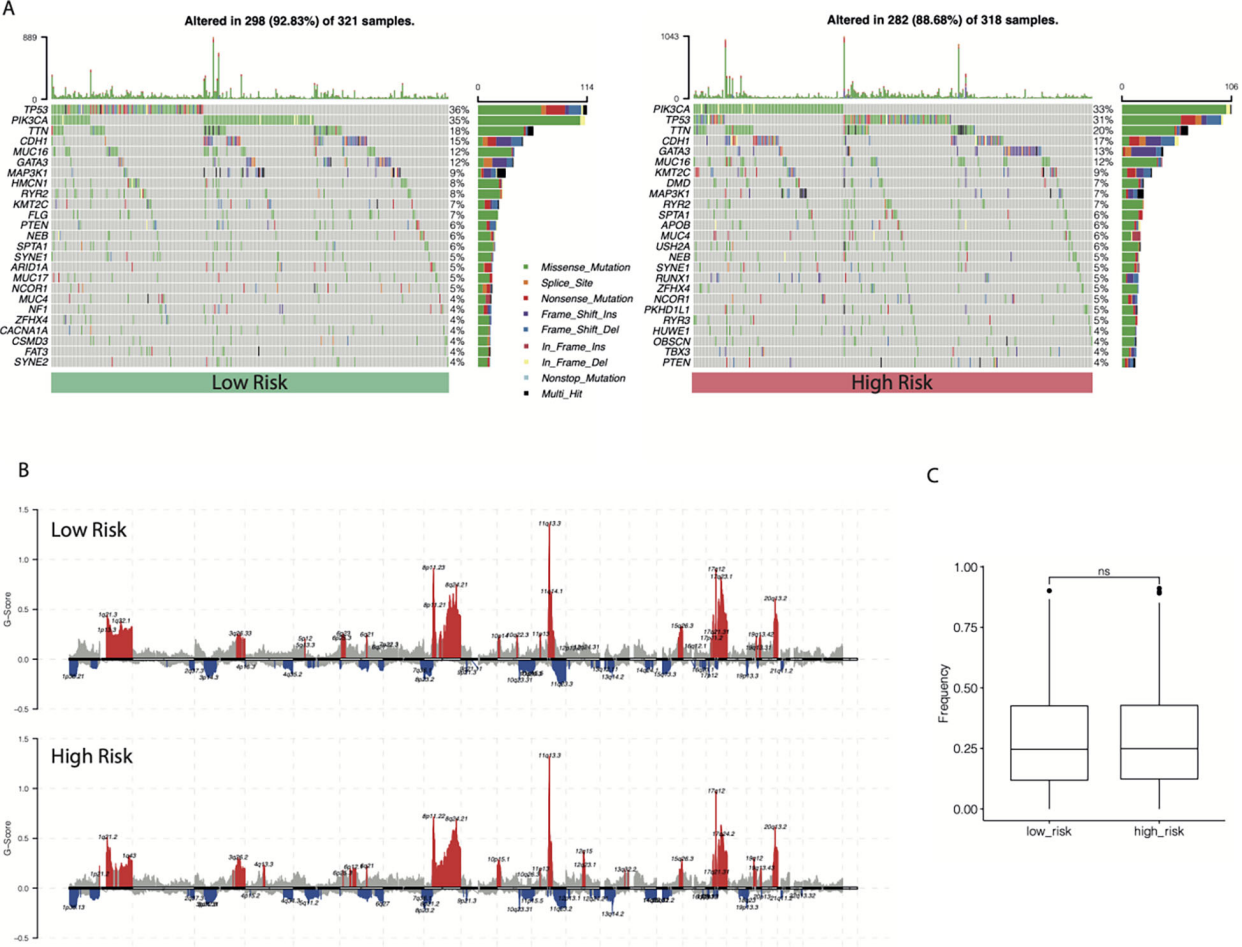
The landscape of SNV and CNV between different cr-TILCD8TSig subgroups. **(A)** Top 25 significantly mutated genes were illustrated in the two subgroups. **(B)** Comparison of significant copy number amplifications and deletions between the two subgroups. **(C)** Comparison of the proportion of fragments with significant copy number variation to the entire genome.

The SNV profiles of the TCGA-BRCA database were also obtained to further explore the mutational landscape in different subgroups. The top 25 genes with the highest mutation rates in the cr-TILCD8Sig-low and -high subgroups are shown in **Figure 11A**. In comparison, we found that the cr-TILCD8Sig-high subgroup exhibited higher mutation counts than the cr-TILCD8Sig-low subgroup. In addition, mutations in *PIK3CA, DMD*, and *APOB* were more common in the cr-TILCD8Sig-high subgroup than in the cr-TILCD8Sig-low subgroup. At the CNV level, there was significantly higher copy number amplification at 4q13.3, 10p15.1, and 12q in the cr-TILCD8Sig-high subgroup, compared with the low-score group, while no significant difference was observed in copy number loss. On chromosome eight, the high-risk group exhibited more and broader copy number amplification events than the low-risk group (**Figure 11B**). Next, we counted the proportion of fragments with significant CNV in the entire genome. No significant difference was found between the high- and low-risk groups in terms of the proportion of CNV fragments (**Figure 11C**).

### Benefits of chemotherapy and immunotherapy in different cr-TILCD8TSig subgroups

**Figure 12:**
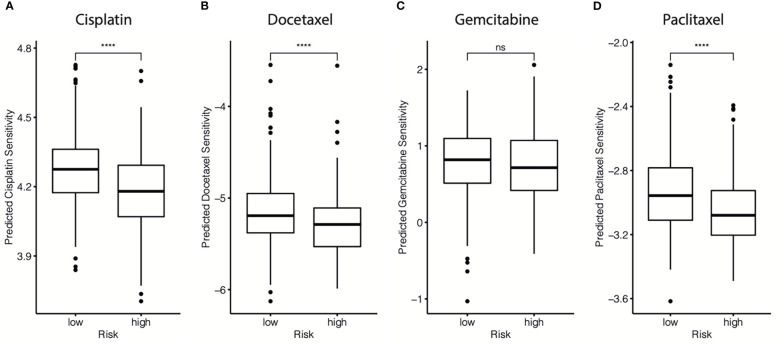
Drug resistance predictions in different cr-TILCD8TSig subgroups. Treatment response of cisplatin **(A)**, doxorubicin **(B)**, gemcitabine **(C)**, and paclitaxel **(D)** were estimated and compared in the GSE25066 cohort. Values were considered statistically significant at p<0.05 (****, p<0.0001).

The chemotherapy information of the GSE25066 database was extracted, and chemotherapy sensitivity to the four most commonly used chemotherapeutic drugs in breast cancer treatment (paclitaxel, cisplatin, docetaxel, and gemcitabine) was estimated using the “pRRophetic” package. We found that the cr-TILCD8TSig-high subgroup exhibited significantly lower sensitivity to cisplatin, docetaxel, and paclitaxel, compared with the cr-TILCD8TSig-low subgroup. Furthermore, a consistent trend was observed in the results of gemcitabine, although no statistically significant differences were observed between the two subgroups (**Figure 12**).

These findings imply that cr-TILCD8TSig-low patients may exhibit a better response to chemotherapy than cr-TILCD8TSig-high patients. These results are consistent with those shown in **Figure 7**, indicating that the cr-TILCD8TSig scoring model exhibited good agreement with chemotherapy sensitivity.

**Figure 13:**
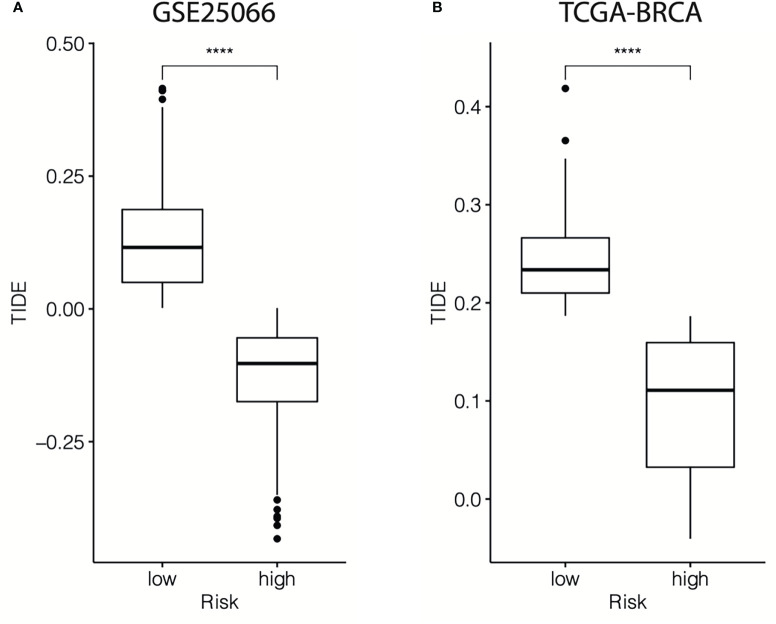
TIDE scores for different cr-TILCD8TSig subgroups in the GSE25066 and TCGA-BRCA datasets. Values were considered statistically significant at p<0.05 (****, p<0.0001).

Then, the TIDE algorithm was used to assess the potential response to immunotherapy in different cr-TILCD8TSig subgroups. To our knowledge, a higher TIDE score implies a higher chance of antitumor immune evasion, thereby predicting a lower response rate to immunotherapy. We found that in the GSE25066 cohort, the TIDE score was dramatically higher in the cr-TILCD8TSig-low subgroup compared with that in the cr-TILCD8TSig-high subgroup (**Figure 13A**). Furthermore, we observed a consistent trend in the TCGA-BRCA cohort (**Figure 13B**). These results revealed that the cr-TILCD8TSig high-risk score indicated resistance to immunotherapy. Interestingly, considering that cr-TILCD8TSig contained the features of T cell exhaustion, these findings were consistent with previous knowledge that T cell exhaustion is closely related to immunotherapy resistance.

### Validation of the predictive nomogram for breast cancer patients

**Figure 14:**
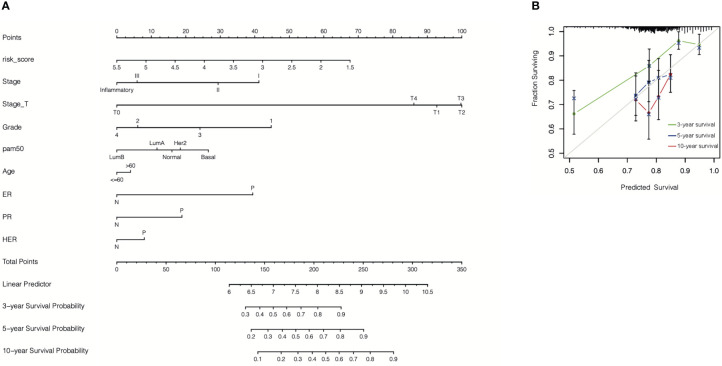
Construction of nomogram. **(A)** A nomogram was constructed to predict 3-, 5-, or 10-year survival. **(B)** Calibration curves of the nomogram for predicting 3-, 5-, or 10-year survival.

Finally, an accurate nomogram containing the cr-TILCD8TSig scoring model and multiple clinical factors, such as stage, stage_T, grade, pam50, age, ER status, PR status, and HER status, was developed using patient data from the GSE25066 cohort (**Figure 14A**). The calibration curves indicated that the 3-, 5-, and 10-year OS could be estimated with a high predictive accuracy (**Figure 14B**).

## Discussion

Extensive evidence has shown that the complexity of the TME constituents contributes to diverse therapeutic responses and various clinical outcomes in breast cancer. Consequently, developing a chemoresistance-related TME classifier of breast cancer for risk stratification and therapeutic decision making has significant clinical implications. In this study, we developed a computational framework (named cr-TILCD8TSig), which was characterized as a risk scoring model of TILCD8Ts, to predict prognosis and chemotherapy responses of breast cancer by comprehensively comparing the TILs, DEGs, and clinical profiles in chemoresistant and chemosensitive cohorts based on machine learning. We proved that the cr-TILCD8TSig scoring model acts as an independent survival predictor and that it has a good discrimination ability in different independent datasets. In addition, we demonstrated that the cr-TILCD8TSig scoring model significantly improved the predictive power of chemotherapy and immunotherapy outcomes.

Recently, TILs been widely accepted to play important roles in regulating tumorigenesis, progression, and treatment response in breast cancer (6, 7). As one of the key components of TILs, TILCD8Ts are considered to be an independent prognostic predictor in breast cancer (8). In addition, with the development and advancement of immunotherapy in breast cancer treatment, the role of TILCD8Ts in immunotherapy has become a focus of breast cancer research. However, chemoresistance remains a major obstacle to effective breast cancer treatment (2, 3). The application potential of the TILCD8T signature for predicting chemotherapy response remains largely undetermined. Thus, in the present study, we focused on studying the correlation between TILCD8T and chemotherapy outcomes. By extracting the mRNA expression patterns of 508 biopsy breast cancer samples before neoadjuvant chemotherapy from the training cohort (GSE25066) and 892 effective samples from the validation cohort (TCGA-BRCA), we found that chemoresistant samples exhibited a higher TILCD8T proportion than chemosensitive samples. This result seems to contradict those of most previous studies. By carefully analyzing our data, we identified three possible reasons:

First, the quantified methods of TIL-CD8+ T cells performed in most previous studies were immunohistochemical analyses (9), whereas in this work, we studied RNA-seq data from nearly 1500 samples, which is more advantageous than the former. Second, previous studies have mostly focused on how TIL-CD8+ T cells influence clinical prognosis or immunotherapy outcomes (17, 18), which is different from the present work, which started from the perspective of identifying cr-TILCD8Ts in breast cancer for chemotherapy stratification. Third, views of cellular heterogeneity in TIL-CD8+ T cell populations, including activated, expanded, and Tex, are extensively accepted (10, 19). Recent advances in single-cell sequencing technology have refined our understanding of Tex, revealing the dysfunctional progression of T lymphocytes, which induces poor clinical outcomes in immunotherapy (20). Interestingly, by comparing the TILs components in chemoresistant and chemosensitive breast cancer, we revealed that the proportions of CD8 T cells dramatically decreased in chemosensitive breast cancer samples than in chemoresistant groups. Based on this, we further extract a series of prognosis related upregulation genes in TIL-CD8 T cells by comparing with other immune cells. Finally, we focused on seven genes, *TCF7, RARRES3, ARL4C, ITK, CDH3, GZMB*, and *KLRD1* to develop a cr-TILCD8TSig. We found interesting that the 7 genes contains 4 (*TCF7, KLRD2, GZMB*, and *ITK*) proven CD8+ T cell exhaustion (Tex) related genes: At the four developmental stages of Tex, the *TCF7, KLRD1*, and *GZMB* have been proved to be a key marker of progenitor 1 (Tex^prog1^), Tex^prog3^, and Tex^prog4^, separately (10). A pan-cancer TILs single-cell RNA-sequencing study including 21 different human cancers also indicated that the development of Tex is tightly regulated by *TCF7* (16). Moreover, Isabelle et al. suggested that *ITK* could be indirectly inhibited by ibrutinib, thus reversing the CD8 T cell exhaustion program (21). This also indicates that *ITK* is correlated with Tex. In this study, the TIDE score was also observed to be dramatically higher in the cr-TILCD8TSig-low subgroup compared with that in the cr-TILCD8TSig-high subgroup in the training and validation cohorts, implying a lower response rate to immunotherapy in the cr-TILCD8TSig-high subgroup than in the -low subgroup. This evidence indicates that cr-TILCD8Ts preferentially exhibit the characteristics of Tex and are in different developmental stages. What’s more, these results implied the feasibility of cr-TILCD8TSig applying in predicting chemoresistant breast cancer.

Accumulating evidence suggests that Tex plays a critical role in immunotherapy resistance, leading to poor clinical outcome (22–24). However, the influence of Tex on chemotherapy outcomes remains unknown. In this study, we demonstrated that a higher TILCD8T proportion indicated chemoresistant breast cancer, whereas a lower TILCD8T proportion indicated chemosensitive breast cancer. Based on this, we constructed a Tex-related TILCD8T signature, cr- TILCD8TSig, to predict chemotherapy response in breast cancer.

In total, our work yielded a computational framework based on integrating CD8+ T cell-related genes, infiltration features of breast cancer chemoresistance-related TILCD8T cells, and clinical profile analysis to identify the specific expression patterns of chemoresistance-correlated TILCD8Ts (named “cr-TILCD8TSig”). In addition, we systematically explored the molecular characteristics, genetic variants, chemotherapy and immunotherapy response features, and the potential clinical application of cr-TILCD8TSig.

This study had several limitations. Due to the limited number of cells obtained from breast cancer biopsy specimens before neoadjuvant chemotherapy, we could not further validate how cr-TILCD8TSig influences chemoresistance using single-cell transcriptomics or proteomics. However, this work prompted us to explore if and how Tex influences chemotherapy outcomes in our next study. Furthermore, our results suggest that patients in the cr-TILCD8TSig-low subgroup may be more responsive to chemotherapy compared to those in the -high group, which needs to be tested in future clinical trials.

## Data availability statement

The datasets presented in this study can be found in online repositories. The names of the repository/repositories and accession number(s) can be found in the article/**Supplementary Material**.

## Author contributions

DQC, JC, MS and YL conceived the theme of the study. DQC and DC conducted the literature search, JC, DQC and XC contributed to data extraction and data analysis. DQC and YZ wrote the original manuscript. JC and ZJ checked and modified the manuscript. All authors contributed to the article and approved the submitted version.
