# Supplementary material for: Construction and validation of chemoresistance-associated tumor- infiltrating exhausted-like CD8+ T cell signature in breast cancer: cr-TILCD8TSig
Source: Front Immunol. 2023 Mar 6;14:1120886. doi: 10.3389/fimmu.2023.1120886 (PMC10025395; doi:10.3389/fimmu.2023.1120886)
Supplement: Supplementary file 1 [file DataSheet_1.docx]

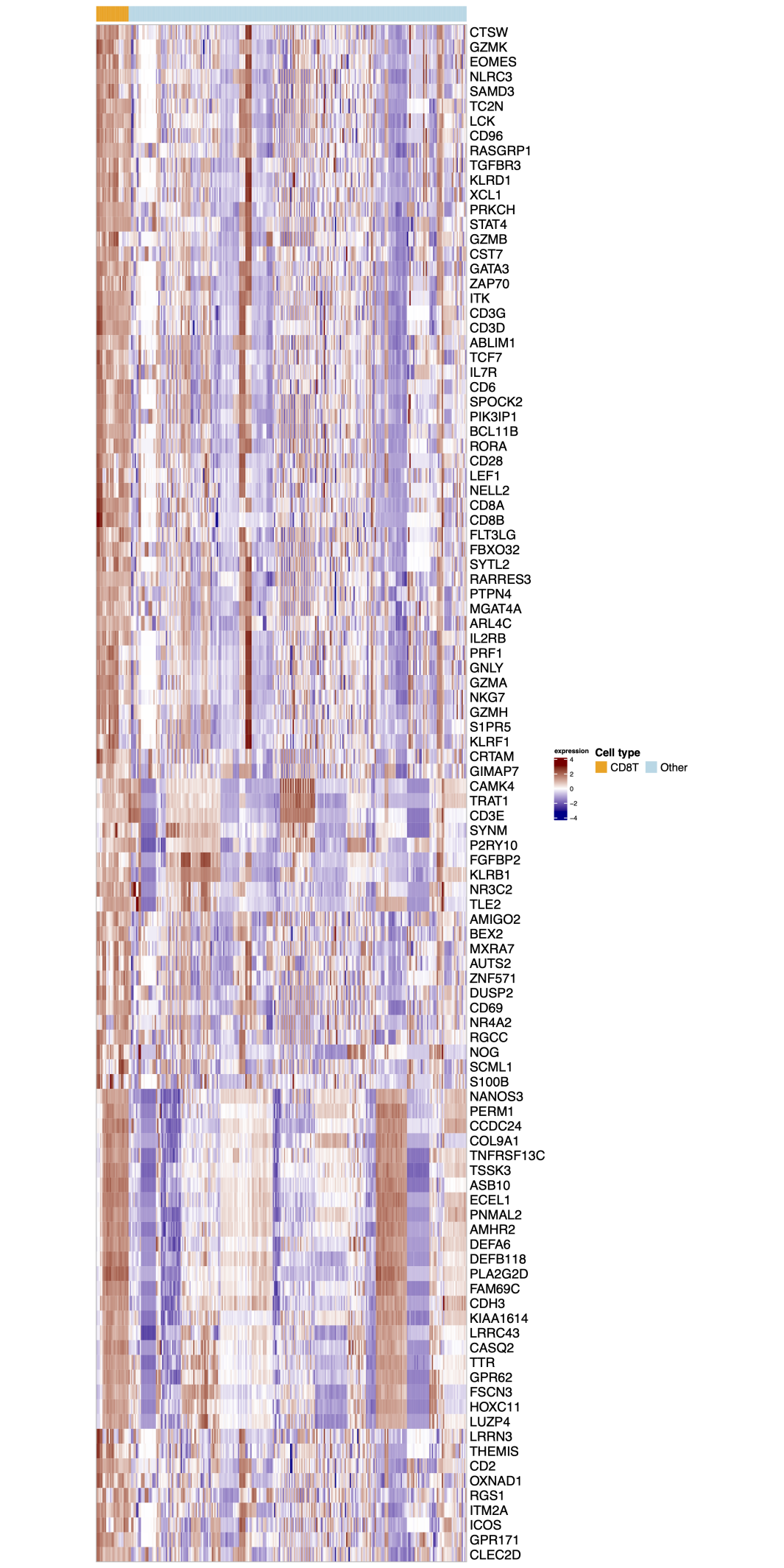


Figure S1. Heat map of specific high-expression genes for CD8T and other types of immune cells.


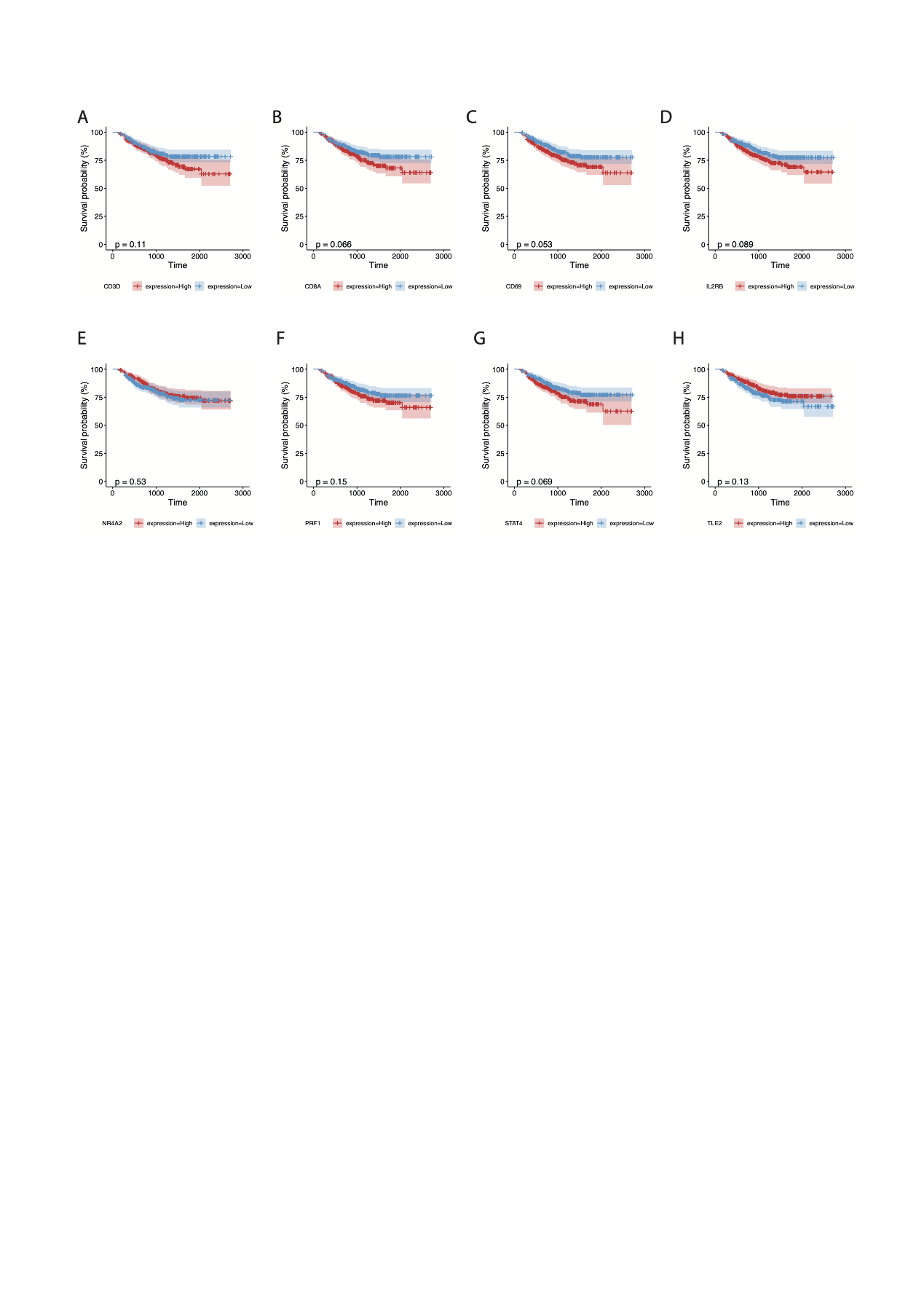


Figure S2. K-M analysis of eight CD8T-specific highly expression genes that did not have significant DRFS effects.
