# Supplementary material for: Construction and validation of chemoresistance-associated tumor- infiltrating exhausted-like CD8+ T cell signature in breast cancer: cr-TILCD8TSig
Source: Front Immunol. 2023 Mar 6;14:1120886. doi: 10.3389/fimmu.2023.1120886 (PMC10025395; doi:10.3389/fimmu.2023.1120886)
Supplement: Supplementary file 2 [file DataSheet_2.docx]

The raw data and code files were share on Jianguoyun/Nutstore, the shareable link as:

https://www.jianguoyun.com/p/DYica1oQ9a-kCxitoe4EIAA
