# Supplementary material for: Construction and validation of chemoresistance-associated tumor- infiltrating exhausted-like CD8+ T cell signature in breast cancer: cr-TILCD8TSig
Source: Front Immunol. 2023 Mar 6;14:1120886. doi: 10.3389/fimmu.2023.1120886 (PMC10025395; doi:10.3389/fimmu.2023.1120886)
Supplement: Supplementary file 4 [file Table_2.docx]

Table S2. Clinical information statistics of GSE25066 samples.

| Clinical Features | | counts |
| --- | --- | --- |
| Age |  |  |
|  | <=60 | 412 |
|  | >60 | 96 |
| Grade |  |  |
|  | 1 | 32 |
|  | 2 | 180 |
|  | 3 | 259 |
|  | 4 | 15 |
|  | NULL | 22 |
| Stage |  |  |
|  | I | 8 |
|  | II | 272 |
|  | III | 224 |
|  | Inflammatory | 4 |
| Stage_T |  |  |
|  | T0 | 3 |
|  | T1 | 30 |
|  | T2 | 255 |
|  | T3 | 145 |
|  | T4 | 75 |
| ER |  |  |
|  | I | 4 |
|  | N | 205 |
|  | NULL | 2 |
|  | P | 297 |
| PR |  |  |
|  | I | 5 |
|  | N | 258 |
|  | NULL | 2 |
|  | P | 243 |
| HER |  |  |
|  | I | 4 |
|  | N | 485 |
|  | NULL | 13 |
|  | P | 6 |
| pam50 |  |  |
|  | Basal | 189 |
|  | Her2 | 37 |
|  | LumA | 160 |
|  | LumB | 78 |
|  | Normal | 44 |
