# Supplementary material for: Construction and validation of chemoresistance-associated tumor- infiltrating exhausted-like CD8+ T cell signature in breast cancer: cr-TILCD8TSig
Source: Front Immunol. 2023 Mar 6;14:1120886. doi: 10.3389/fimmu.2023.1120886 (PMC10025395; doi:10.3389/fimmu.2023.1120886)
Supplement: Supplementary file 8 [file Table_6.docx]

Table S6. List of genes significantly associated with prognosis in Cox univariate analysis.

| **Tags** | **beta** | **HR** | **CI_lower** | **CI_upper** | **(95%_CI_for_HR)** | **wald.test** | **p.value** |
| --- | --- | --- | --- | --- | --- | --- | --- |
| CDH3 | 0.17 | 1.2 | 1.1 | 1.3 | (1.1-1.3) | 14 | 0.00015 |
| ARL4C | 0.38 | 1.5 | 1.2 | 1.8 | (1.2-1.8) | 12 | 0.00042 |
| RARRES3 | -0.21 | 0.81 | 0.71 | 0.91 | (0.71-0.91) | 12 | 0.00062 |
| CD69 | 0.26 | 1.3 | 1.1 | 1.5 | (1.1-1.5) | 10 | 0.0012 |
| KLRD1 | 0.37 | 1.4 | 1.1 | 1.8 | (1.1-1.8) | 9.5 | 0.002 |
| ITK | 0.3 | 1.4 | 1.1 | 1.6 | (1.1-1.6) | 9 | 0.0026 |
| CD8A | 0.29 | 1.3 | 1.1 | 1.6 | (1.1-1.6) | 7.9 | 0.0049 |
| TLE2 | -0.22 | 0.8 | 0.69 | 0.94 | (0.69-0.94) | 7.8 | 0.0051 |
| TCF7 | 0.31 | 1.4 | 1.1 | 1.7 | (1.1-1.7) | 7.8 | 0.0052 |
| CD3E | 0.32 | 1.4 | 1.1 | 1.7 | (1.1-1.7) | 7.4 | 0.0063 |
| STAT4 | 0.38 | 1.5 | 1.1 | 1.9 | (1.1-1.9) | 7.3 | 0.0067 |
| PRF1 | 0.23 | 1.3 | 1.1 | 1.5 | (1.1-1.5) | 7.3 | 0.0068 |
| GZMB | 0.21 | 1.2 | 1.1 | 1.4 | (1.1-1.4) | 7.2 | 0.0072 |
| CD2 | 0.22 | 1.3 | 1.1 | 1.5 | (1.1-1.5) | 7.1 | 0.0075 |
| GZMA | 0.24 | 1.3 | 1.1 | 1.5 | (1.1-1.5) | 6.4 | 0.011 |
| CD3D | 0.18 | 1.2 | 1 | 1.4 | (1-1.4) | 5.9 | 0.015 |
| SPOCK2 | 0.29 | 1.3 | 1.1 | 1.7 | (1.1-1.7) | 5.6 | 0.018 |
| CD6 | 0.22 | 1.2 | 1 | 1.5 | (1-1.5) | 5.5 | 0.019 |
| LRRN3 | 0.28 | 1.3 | 1 | 1.7 | (1-1.7) | 5.4 | 0.021 |
| IL2RB | 0.32 | 1.4 | 1 | 1.8 | (1-1.8) | 5.2 | 0.023 |
| NR4A2 | -0.2 | 0.82 | 0.68 | 1 | (0.68-1) | 3.9 | 0.048 |
