# Supplementary material for: Construction and validation of chemoresistance-associated tumor- infiltrating exhausted-like CD8+ T cell signature in breast cancer: cr-TILCD8TSig
Source: Front Immunol. 2023 Mar 6;14:1120886. doi: 10.3389/fimmu.2023.1120886 (PMC10025395; doi:10.3389/fimmu.2023.1120886)
Supplement: Supplementary file 9 [file Table_7.docx]

Table S7. K-M analysis of the 21 genes.

| **gene** | **HR** | **95%CI-lower** | **95%CI-upper** | **Cindex** | **logtestp** | **cutoff** |
| --- | --- | --- | --- | --- | --- | --- |
| KLRD1 | 0.43 | 0.29 | 0.65 | 0.59051332 | 3.50E-05 | 7.91175863 |
| ARL4C | 0.53 | 0.35 | 0.79 | 0.57441196 | 0.0015 | 9.52455886 |
| CD6 | 0.54 | 0.36 | 0.8 | 0.56978558 | 0.002 | 8.95563543 |
| GZMA | 0.56 | 0.37 | 0.83 | 0.57472385 | 0.0034 | 8.39883412 |
| CD2 | 0.56 | 0.38 | 0.83 | 0.56235218 | 0.0035 | 9.58781129 |
| SPOCK2 | 0.59 | 0.4 | 0.88 | 0.56578298 | 0.0091 | 9.31976477 |
| CD3E | 0.6 | 0.4 | 0.88 | 0.55782976 | 0.0092 | 8.84678748 |
| CDH3 | 0.62 | 0.42 | 0.91 | 0.58359974 | 0.015 | 7.91281397 |
| RARRES3 | 1.6 | 1.1 | 2.4 | 0.57250162 | 0.014 | 10.8350662 |
| GZMB | 0.62 | 0.42 | 0.92 | 0.55711501 | 0.016 | 8.02039884 |
| LRRN3 | 0.63 | 0.42 | 0.93 | 0.55922027 | 0.019 | 8.27775355 |
| TCF7 | 0.65 | 0.44 | 0.97 | 0.54482131 | 0.032 | 10.7585108 |
| ITK | 0.67 | 0.45 | 0.99 | 0.54063678 | 0.045 | 8.50604991 |
| CD69 | 0.68 | 0.46 | 1 | 0.54667966 | 0.053 | 7.4954113 |
| CD8A | 0.7 | 0.47 | 1 | 0.53581546 | 0.066 | 9.2974308 |
| STAT4 | 0.7 | 0.47 | 1 | 0.5411566 | 0.069 | 9.0136467 |
| IL2RB | 0.71 | 0.48 | 1.1 | 0.53790773 | 0.089 | 9.81074702 |
| CD3D | 0.73 | 0.49 | 1.1 | 0.52652372 | 0.11 | 9.6110773 |
| TLE2 | 1.3 | 0.91 | 2 | 0.53879142 | 0.13 | 8.23525803 |
| PRF1 | 0.75 | 0.51 | 1.1 | 0.53134503 | 0.15 | 8.89887693 |
| NR4A2 | 1.1 | 0.77 | 1.7 | 0.52330084 | 0.53 | 8.03173207 |
